# Understanding client satisfaction with a health insurance scheme in Nigeria: factors and enrollees experiences

**DOI:** 10.1186/1478-4505-9-20

**Published:** 2011-05-25

**Authors:** Shafiu Mohammed, Mohammad N Sambo, Hengjin Dong

**Affiliations:** 1 Institute of Public Health, Medical Faculty Heidelberg, Heidelberg University, Im Neuenheimer Feld 324, 69120, Heidelberg, Germany; 2 Faculty of Medicine, Department of Community Medicine, Ahmadu Bello University, P.M.B 1044, Zaria, Nigeria; 3 Center for Health Policy Studies, Zhejiang University Medical School, Hangzhou 310058, P.R. China

## Abstract

**Background:**

Health insurance schemes have been widely introduced during this last decade in many African countries, which have strived for improvements in health service provision and the promotion of health care utilization. Client satisfaction with health service provision during the implementation of health insurance schemes has often been neglected since numerous activities take place concurrently. The satisfaction of enrollees and its influencing factors have been providing evidence which have assisted in policy and decision making. Our objective is to determine the enrollee's satisfaction with health service provision under a health insurance scheme and the factors which influence the satisfaction.

**Methods:**

This retrospective, cross-sectional survey took place between May and September 2008. Two hundred and eighty (280) enrollees insured for more than one year in Zaria-Nigeria were recruited using two stage sampling. Enrollee's satisfaction was categorized into more satisfied and less satisfied based on positive responses obtained. Satisfaction, general knowledge and awareness of contribution were each aggregated and assessed as composite measure. Logistic regression analysis was used to analyze factors that influenced the satisfaction of enrollees.

**Results:**

A high satisfaction rate with the health insurance scheme was observed (42.1%). Marital status (p < .05), general knowledge (p < .001) and awareness of contributions (p < .05) positively influenced clients' satisfaction. Length of employment, salary income, hospital visits and duration of enrolment slightly influenced satisfaction.

**Conclusions:**

This study highlighted the potential effects of general health insurance knowledge and awareness of contributions by end-users (beneficiaries) of such new program on client satisfaction which have significant importance. The findings provided evidence which have assisted the amendment and re-prioritization of the medium term strategic plan of operations for the scheme. Future planning efforts could consider the client satisfaction and the factors which influenced it regularly.

## Background

Health insurance as a complementary or alternative source of health care financing has become important in the developing world [1]. It has been implemented as part of health reform programmes and strategies aimed towards providing effective and efficient health care for citizens, most especially for the poor and vulnerable [1]. Health insurance schemes in many low and middle income countries (LMICs), most especially in the African continent, are still in their early stages of implementation with the goal of universal coverage of the population. In Nigeria, health insurance has been introduced to the populace in mid 2005. The first phase has been the implementation of the formal-sector programme, which began four and half years ago. There has been a need to understand the enrolee's satisfaction of health service provision in the health insurance scheme in order to effectively monitor the process, and to improve the scheme's implementation.

Already there has been insufficient knowledge and awareness of the health insurance activities by those enrolled in the scheme. Complaints have arisen where providers denied enrollees their full entitlements and some providers have charged additional fees on the pretext of non-inclusion of the service in the benefit package [2]. Again, Insured-persons have complained of poor attitude and behaviour of service providers operating in the health insurance scheme [2]. Assessing the appropriateness of care and clients satisfaction is crucial to have assured the continuous attractiveness of the care contracted [3]. There has been insufficient literature in LMICs, most notably in African countries that dealt with clients satisfaction based on the knowledge and awareness of enrollees within a health insurance scheme setting. One of the very few studies that dealt with enrollee's experiences and concerns in a West African region pointed out that research of clients satisfaction should be ongoing [3].

Certainly, problems associated with health service provision needed to be understood and rapidly resolved at all times. This would have helped immensely in the future implementation strategies of the scheme by identifying what has happened, and how to progress to make it better for all. It would aid to have improved the monitoring of health care providers' activities within the scheme. Like any other country, the national health insurance scheme (NHIS) in Nigeria aimed to provide health insurance so that insured persons and their dependants are able to have access to good quality and cost-effective healthcare services [4]. But, the formal sector programme of the NHIS specified that "contributions made by or for an insured person entitled him or her, a spouse and four biological children under the age of 18 years to a defined health benefits package" [4]. The Formal Sector Social Health Insurance Programme is a "scheme where the healthcare of employees in the formal sector is paid from funds created by pooling the contributions of employees and or employers" [4]. Currently, only the public sector (federal employees), a subset of the formal sector group has been actively participating.

It is essential to consider how policy interventions such as the NHIS have influenced the barriers to health care utilization, which not only included geographical access, but also perceived quality of care and access to information [5]. Measures of health system effectiveness should be aligned with improvements in access to quality of care and client satisfaction [6]. Satisfaction surveys have been widely used to address the problems of access and performance [6-9]. Indeed, they have been instrumental in helping government agencies to identify target groups, clarify objectives, define measures of performance, and develop performance information systems [10,11]. Supportively, patient satisfaction is a dominant concern that is intertwined with strategic health services decisions [12,11].

The focus on client satisfaction with the health insurance scheme's service provision after more than four years of active implementation highlights the importance of monitoring and evaluation. It provides information regarding the activities of the health care providers participating in the health insurance scheme. Client satisfaction of any health system or health insurance scheme has been, in most cases, associated with certain individual characteristics and factors, which encompass socio-economic, demographic and cultural factors. However, enrollee's knowledge and awareness of the health insurance system are often not taking into consideration. Policy and decision makers have to understand the potential factors influencing enrollee's satisfaction in order to viably implement such health insurance schemes. Here, we focussed on client satisfaction with health service provision and factors that could have influenced the enrollee's satisfaction. This study was carried out to monitor the progress of implementation of the health insurance scheme in terms of health service provision.

Our hypothesis was that an insured person (household head) would either be more satisfied or less satisfied with the scheme based on certain individual factors and encounter with health care providers (HCPs). We assumed that the aged, females, and low salary income earners were expected to be more satisfied. Enrollees with longer years of service, poorer health condition, frequent hospital visits and longer duration of enrolment in the health insurance scheme were also expected to be more satisfied. We have also explored religion and native language differences. Individuals with a higher educational level, high occupational status, polygamous status, more than four children and residence outside the city were expected to be less satisfied. The most important hypothesized factors were that enrollees with better general knowledge of the health insurance scheme and awareness of monetary contributions were expected to be more satisfied. This hypothesis in respect to the assumptions was supported by the theoretical framework of various satisfaction reviews and adaptations [13-16].

## Methods

### Study area and population

A cross-sectional survey was conducted in Zaria at Ahmadu Bello University located on the high plains of Northern Nigeria, 270 Km northwest of Abuja, the country's capital. Zaria is semi-urban and it has both public and private health facilities. The health facilities are spread around the metropolis and its environs. The University has two campuses, 14 faculties and 82 academic departments [17]. The study population was the staff of the university enrolled in the formal sector programme of the health insurance scheme in Nigeria. The study site was chosen because it has one of the largest enrollees in the health insurance scheme as identified by the regulatory agency. The study took place between May and September 2008. At that time, the total staff population was 7800 (both academic and non academic) [17]. Two thousand four hundred and fifty eight (2458) staff are found to be fully enrolled in the health insurance scheme. This study protocol was approved by the University Research Ethics Committee ABU-Nigeria (VC/P. 18890) and also the Ethics Commission of Heidelberg University, Germany.

### Sampling

Two-stage sampling method was used to select the study subjects. We employed simple random sample selection at each stage to eliminate selection bias. The first stage involved selecting seven out of fourteen faculties. These seven faculties included 36 departments. The second stage involved the selection of staff from each of the 36 departments using proportionate method based on the size of the departments. Only insured persons enrolled for more than one year in the health insurance scheme are included in the study. Verification of enrolment was carried out in collaboration with the NHIS desk officers in Zaria-Nigeria. The participants had to have met the inclusion criteria at both stages to be eligible for the study.

A minimum sample size of 250 for this study was estimated. We calculated the sample size using a *power *of 90% at a *(2-sided) *significance level of 5% based on an assumed proportion of (.5) and a null hypothesis value of (.4) with detectable difference of 10. The detectable difference was taken into consideration which dealt with the assumed knowledge gap that could have existed in the population. The sample size was calculated using the "single proportion formula for sample size determination" method [18]. To allow for non-response and other possible incidentals, we chose to increase the sample size to 300 and then obtained 280 given the stated response rate.

### Questionnaire

A pre-tested interviewer-administered questionnaire was used. It was addressed to insured-persons who had been enrolled in the health insurance scheme for at least one year. The questionnaire included five sections with questions related to the socio-economic and demographic characteristics of the respondents, general knowledge of the health insurance scheme, awareness of monetary contributions, utilization of health services, and clients' satisfaction. Three hundred questionnaires were distributed.

### Definitions of clients' satisfaction; general knowledge of health insurance; and awareness of monetary contributions

Clients' satisfaction was divided into two categories. Firstly, enrollees who had positive responses on any four or more of the six criteria, which formed the composite index measure, were defined as more satisfied. Secondly, those with zero to three positive responses were defined as less satisfied. The composite index measure comprised of criteria which concerned (i) the receipt of courteous attention at the hospital, (ii) decreased waiting times, (iii) the availability of a doctor or nurse throughout the hospital visits, (iv) the receipt of better health care, (v) the frequency of hospital visits when sick, and (vi) the preference for health insurance in participants' own neighbourhoods. The framework for these dimensions was derived from satisfaction reviews and adaptation of previous measures [15,13,14].

General knowledge as a composite was based on whether the respondents felt that the health insurance was a good way to have helped them in solving their health expenditure problems. It also included knowledge of the basic benefits package of the health insurance scheme.

Awareness of monetary contribution as a composite focussed on whether the respondents were knowledgeable of both the employees' and the employer's monetary contributions to the health insurance programme.

### Statistical analysis

To analyze client satisfaction, enrollees were categorized into the above mentioned two groups i.e. more satisfied and less satisfied individuals. The explanatory variables selected were based on the study hypotheses. We assessed bivariate associations between all variables and client satisfaction. Differences between the associated explanatory variables subsequently were analyzed by comparing means using t-tests and chi-square tests.

After testing for collinearity, we eliminated only one variable with a correlation above (.75) and created our final model. The eliminated variable was health condition (people who were sick or not). It was strongly correlated with hospital visits (people who had consulted a doctor or not). We kept the utilization variable because of its relevance to the study since health insurance aimed to improve and promote utilization of health services. Logistic regression was used to model the potential factors associated with clients' satisfaction. A *P*-value of (.05) was used as the threshold for statistical significance. The SPSS program (SPSS^® ^17.0, 2008; Spss Inc., Wacker Drive, Chicago, USA) was used for all analyses.

## Results

The response rate was 93.33% (i.e 280 insured-persons responded). The socio-demographic characteristics of the sample are presented in table 1. The mean age (in years) of the whole sample in the study was 42.49 (SD = 8.97). Older people (mean = 44.42) reported more satisfaction than young people (mean = 41.09) (p = .001). Both males and females showed no significant difference in satisfaction (p = .911). Mean duration of service (in years) at the institution was 14.47 (SD = 9.84) and a median of 15.00. Respondents with longer length of employment (mean = 17.49) were more satisfied, than those with shorter length of employment (mean = 12.27). The mean income of respondents was (USD) $644.40 (SD = 391.17). There was no significant difference in satisfaction among respondents' religion (p = .553) and ethnic group (p = .423). The majority of respondents had received tertiary education. Respondents with a tertiary level of education 128 (61.0%) were less satisfied, while those below the tertiary level 37 (52.0%) were more satisfied. Most of the senior staff 121 (62.0%) were less satisfied, while the junior staff 43 (51.0%) were more satisfied. Respondents with polygamous status were more satisfied compared to those with non-polygamous status. The majority of those that were widowed, divorced, or single 22 (96.0%) were less satisfied than those with either polygamous or monogamous status.

**Table 1:** Socio-demographic characteristics of sample by "Client Satisfaction"

Variables	Total (%)	More Satisfied (%)	Less Satisfied (%)	*P-value*
Total cases	280 (100)	118 (42.1)	162 (57.9)	
Sex:				.911
Male	231 (100)	97 (42.0)	134 (58.0)	
Female	49 (100)	21 (43.0)	28 (57.0)	
Age (yrs)	42.49	44.42	41.09	.001
Length of employment (yrs)	14.47	17.49	12.27	.001
Religion:				.553
Muslim	160 (100)	65 (41.0)	95 (59.0)	
Christian	120 (100)	53 (44.0)	67 (56.0)	
Ethnic group:				
Hausa	118 (100)	53 (45.0)	65 (55.0)	.423
Others	162 (100)	65 (40.0)	97 (60.0)	
Residence:				.206
Inside city	150 (100)	58 (39.0)	92 (61.0)	
Outside city	130 (100)	60 (46.0)	70 (54.0)	
Educational level:				
Tertiary	209 (100)	81 (39.0)	128 (61.0)	.049
Below Tertiary	71 (100)	37 (52.0)	34 (48.0)	
Occupation status:				.045
Senior Staff	196 (100)	75 (38.0)	121 (62.0)	
Junior Staff	84 (100)	43 (51.0)	41 (49.0)	
Salary income ($)	644.40	613.25	667.09	.256
Marital status:				.001
Polygamy	55 (100)	27 (49.0)	28 (51.0)	
Monogamy	202 (100)	90 (45.0)	112 (55.0)	
Others	23 (100)	1 (4.0)	22 (96.0)	
No. of children:				.080
> 4	109 (100)	53 (49.0)	56 (51.0)	
< = 4	171 (100)	65 (38.0)	106 (62.0)	
				

Note: *t-Test *for age, length of employment, and income. χ*^2^*-Test for other variables.

The insurance and health related responses of the sample are presented in table 2. The respondents reported a significant difference in satisfaction (p = .001) with length of enrolment. Respondents with longer length of enrolment in the insurance 76 (54.0%) were more satisfied, while those with shorter length of enrolment in the insurance 98 (70.0%) were less satisfied. There was a significant difference in satisfaction (p = .001) with respondents' general knowledge of health insurance. The respondents with less knowledge 130 (75.0%) were less satisfied, while those respondents with more knowledge 74 (70.0%) were more satisfied. Again, there was a significant difference in satisfaction (p = .001) with respondents' awareness of money contribution in the health insurance. Those respondents with less awareness 132 (64.0%) were less satisfied, while those respondents with more awareness 45 (70.0%) were more satisfied. There was a significant difference in satisfaction (p = .020) with respondents' health condition. Again, there was a significant difference in satisfaction (p = .007) with respondents' hospital visits.

**Table 2:** Insurance and health related responses of sample by "Client Satisfaction"

Variables	Total (%)	More Satisfied (%)	Less Satisfied (%)	*P-value*
Total cases	280 (100)	118 (42.1)	162 (57.9)	
Length of enrolment:				.001
> = 2 yrs	140 (100)	76 (54.0)	64 (46.0)	
< 2 yrs	140 (100)	42 (30.0)	98 (70.0)	
General Knowledge on health insurance:				.001
More Knowledge	106 (100)	74 (70.0)	32 (30.0)	
Less Knowledge	174 (100)	44 (25.0)	130 (75.0)	
Awareness on money contribution:				.001
More Aware	75 (100)	45 (60.0)	30 (40.0)	
Less Aware	205 (100)	73 (36.0)	132 (64.0)	
Health Condition:				.020
Somebody Sick in past 1 month	170 (100)	81 (48.0)	89 (52.0)	
Nobody Sick in past 1 month	110 (100)	37 (34.0)	73 (66.0)	
Hospital Visit:				.007
Had Visit in past 1 month	159 (100)	78 (49.0)	81 (51.0)	
No Visit in past 1 month	121 (100)	40 (33.0)	81 (67.0)	
				

Note: *t-Test *for age, length of enrollment, and income. χ^*2*^-Test for other variables.

### Satisfaction and factors influencing clients' satisfaction

Less than half of the respondents reported being satisfied (42.1%). All the aforementioned variables were included using logistic regression (Table 3). Marital status (p < .05), general knowledge of health insurance (p < .001), and awareness of monetary contributions (p < .05) significantly influenced enrollee's satisfaction with health service provision in the health insurance scheme. Respondents with polygamous status were more satisfied than others (β= 2.735; SE = 1.188, p = .021). Those with monogamous status were equally more satisfied than others (β= 2.258; SE = 1.117, p = .043). Insured persons with more knowledge of the health insurance scheme were more satisfied than those with less knowledge (β= 2.010; SE = .326, p = .001). Enrollees with more awareness of the monetary contributions in the health insurance were more satisfied than those with less awareness (β= .720; SE = .341, p = .035). Length of employment, salary income, hospital visits and length of enrolment in the health insurance slightly influenced satisfaction.

**Table 3:** Factors influencing "Clients Satisfaction" (logistic regression)

Variables	Description and coding parameter (1)	β	S.E.	P-value	Exp(B)	( 95% CI )	
(Constant)		-4.754	1.416	.001	.009		
Sex	Male = 1, Female = 0	.139	.414	.737	1.149	.511	2.587
Age	Age in years	.015	.030	.610	1.015	.958	1.076
Staff duration	Staff duration of service in years	.042	.025	.091	1.043	.993	1.096
Religion	Muslim = 1, Christian = 0	-.520	.425	.221	.594	.258	1.368
Ethnic group	Hausa = 1, Others = 0	.414	.404	.306	1.513	.685	3.342
Residence	Inside city = 1, Outside city = 0	-.046	.340	.893	.955	.490	1.860
Educational level:	Tertiary = 1, Below Tertiary = 0	-.191	.475	.687	.826	.326	2.095
Occupation status:	Senior staff = 1, Junior staff = 0	.255	.433	.556	1.291	.552	3.019
Salary income ($)	Monthly salary income in US ($) equiv.	.000	.001	.071	.999	.998	1.000
Marital status: Polygamy Vs others	Polygamy = 1, Others = 0	2.735	1.188	.021	15.415	1.502	158.173
Monogamy Vs others	Monogamy = 1, Others = 0	2.258	1.117	.043	9.562	1.071	85.393
No. of children	> 4 children = 1, < = 4 children = 0	-.302	.374	.419	.739	.355	1.539
Hospital Visit in the past 1 month	Had Visit = 1, No Visit = 0	.561	.323	.083	1.752	.930	3.299
Length of enrolment (yrs)	> = 2 years = 1, < 2 years = 0	.516	.314	.100	1.676	.905	3.102
General knowledge on insurance	More knowledge = 1, less knowledge = 0	2.010	.326	.000	7.465	3.944	14.130
Awareness on money contribution	More aware = 1, less aware = 0	.720	.341	.035	2.055	1.054	4.007
							
n		280					
							
Nagelkerke R^2^		.417					
-2 log likelihood		277.188^a^					
Cox & Snell R^2^		.310					

Model test: LR χ*^2^*(16) = 104.031, p < 0.001

## Discussion

The study revealed that clients satisfaction rate with the health insurance scheme was somewhat low. Enrollee's satisfaction of service provision served as an important aid to monitor the progress of implementation activities of the scheme. However, certain factors such as general knowledge of the health insurance scheme and awareness of monetary contributions greatly influenced enrollee's satisfaction of health care delivery. Ways of creating better knowledge of health insurance activities among the population were given top priority by both the policy and decision makers, which previously were insufficient until this stage (middle) of implementation. These formed a major part of considerations in the amended medium term strategic plan of operations of the NHIS.

Older clients were more satisfied with service provision than the younger clients. There is direct relationship between enrollee's satisfaction and age which has been similar with related studies [14,19]. Studies in the developed nations have demonstrated that, the most consistent relationship with service satisfaction are the age and sex of clients [14,19]. Still, in developing nations, variations in marital status had influence on enrollee's satisfaction. The polygamous were more satisfied than the monogamous. It was a contrary to our initial hypothesis which suggested that, enrollees with polygamous status would be less satisfied due to the entitlement of principal beneficiary. Already, the NHIS specified that "contributions made by an insured person entitled him, his spouse and four biological children under the age of 18 years to a defined health benefits package" [4,16]. But, polygamy is a religious and cultural norm in many societies in Nigeria as well as other African countries which required careful consideration. Information on how to extend coverage to other family members might enhance enrollee's satisfaction which has not been readily available. Studies within the country context have shown that, marital status has a significant influence on peoples attitude towards insurance [20]. These findings assisted NHIS policy makers to incorporate the socio-cultural context of marital status into the medium term strategic plan of operations, aimed at improving the dissemination of information to all users of the health insurance scheme on how other extra dependent family members of the insured persons are to be enrolled [21].

Insured persons' knowledge of the health insurance scheme was a vital determinant of perceived satisfaction of health care services. Enrollees knowledge of the health insurance was aggregated to their understanding of insurance to be a good way of helping clients' to relieve their health expenditure problems and also their knowledge of the basic benefits package of the health insurance scheme. Poor knowledge of the benefit package has affected utilization rates of health facilities in developing countries [22,23]. Effective monitoring mechanisms should be implemented to ensure that the benefit package has been used in full by all those who are entitled which can improve their satisfaction. Unless information on benefit package is readily available to enrollees, they may not fully access all services because of lack of understanding of their entitlements [24,25]. Enrollee's poor knowledge of health insurance leads to less satisfaction of health service provision. Enrolee satisfaction improves only if they have good understood of how the health insurance scheme works and knew what has been offered by the scheme. The amended medium term strategic plans has emphasized that health maintenance organizations must collaborate with the regulatory agency in the provision of IEC materials regarding the benefit package offered to enrollees.

Insured persons are more satisfied if they have been aware of the contributions made by both the employees and employer. The less awareness of enrollees with health insurance activities, the less satisfied they become of its offerings in terms of service provision. Better awareness of the enrollees might enhance interactions between patients and health care providers due to better satisfaction of services. There are tendencies that, those who do not have full knowledge of insurance services offered would likely evaluate schemes poorly [26].

The enrollee's satisfaction with service provision was somewhat low. Monitoring of health service provision could assist to provide important information when health insurance satisfaction declines. Regulatory agencies should check the situation regularly so as to avert problems before they become crises. Policymakers and monitors needed to realise that, individual patients recognize an effective health system or health insurance scheme as one that provides timely access to the full array of necessary services, efficacious and safe care leading to improvement in health, continuity of care, and respect [11,26-28].

General knowledge of the health insurance package, awareness of monetary contributions, frequent hospital visits due to illnesses, length of employment and length of enrolment in the health insurance programme confirmed our hypotheses because they positively influenced satisfaction. Conversely, more satisfaction in the polygamous enrollees was contrary to the supposition as previous mentioned. Positive linkages with satisfaction were associated with enrollees who had more knowledge of the health insurance, frequently visited the hospital, had longer length of enrolment, and also had some awareness of monetary contributions. These findings suggest that enrollee's satisfaction with health services provision in the scheme could be influenced by several factors. The factors which lead to less satisfaction could be addressed properly to improve on the health insurance activities. Health care provider's politeness toward clients, decreased hospital waiting times, and increased availability of hospital personnel at all times served as composite measure of satisfaction and will help in improving client satisfaction with service provision in the health insurance scheme.

Despite the population consisted of heterogeneous individuals with different socio-economic and cultural backgrounds, which prevailed across Nigeria. However, a study limitation was whether the findings from this survey could be fully generalized to the various facets of the country. Further similar studies are needed in other settings for comparison due to knowledge gap because the studied population was from an academic setting. The dynamic nature of certain regions of Nigeria might exhibit some socio-demographic differences due to cultural, educational, religious and economic status which needed careful consideration.

## Conclusions and implications

Enrollee's satisfaction with service provision of health insurance can be influenced by several factors especially the poor knowledge of health insurance and lack of awareness of contribution by the insured persons. Periodic identification of related influencing factors on client satisfaction could assist in guiding policy and decision making to detect promising pathways to improve any nascent program like health insurance schemes. Improved knowledge and better awareness of the scheme's activities by the enrollees could be augmented through the provision of requisite available information to the insured persons at all times.

## Competing interests

The authors declare that they have no competing interests.

## Authors' contributions

SM, HD and MNS conceptualized and designed the study protocols. SM and MNS carried out the field study work. SM and HD analysed and interpreted the data. SM and HD drafted the manuscript. HD critically reviewed the findings. All authors read and approved the final manuscript.

## Ethics approval

This study protocol was approved by the University Research Ethics Committee ABU-Nigeria (VC/P. 18890) and also the Ethics Commission of Heidelberg University, Germany.
